# Genome Sequence of *Clostridium butyricum* Strain DSM 10702, a Promising Producer of Biofuels and Biochemicals

**DOI:** 10.1128/genomeA.00563-13

**Published:** 2013-08-01

**Authors:** Bo Xin, Fei Tao, Yu Wang, Chao Gao, Cuiqing Ma, Ping Xu

**Affiliations:** State Key Laboratory of Microbial Technology, Shandong University, Ji’nan, Chinaa; State Key Laboratory of Microbial Metabolism and School of Life Sciences & Biotechnology, Shanghai Jiao Tong University, Shanghai, Chinab

## Abstract

*Clostridium butyricum* strains have been considered promising producers of biofuels and biochemicals, such as hydrogen, butanol, butyric acid, and 1,3-propanediol. Here, we present a 4.59-Mb assembly of the genome sequence of DSM 10702 (VPI 3266), a type strain of *C. butyricum*.

## GENOME ANNOUNCEMENT

Due to the depletion of fossil fuels, environmental concerns, and sustainability issues, the conversion of renewable biomass to valuable fuels and chemicals by microorganisms has drawn global interest (1, 2). Hydrogen (H_2_) is an environmentally safe, renewable energy resource and an ideal clean alternative to fossil fuels (3). Butanol is accepted as a promising biofuel with several advantages (4). Besides bioderived fuels, biomass-converted biochemicals are also attractive. 1,3-Propanediol (1,3-PD) is one of the most important precursors of biomaterials. It is mainly used as a monomer for novel polyester and biodegradable plastics, such as polytrimethylene terephthalate (2).

*Clostridium butyricum* is an anaerobic bacterium that can ferment sugar and glycerol to several biofuels and precursors of biomaterials, such as H_2_, butanol, butyric acid, and 1,3-PD (5–8). Theoretically, *Clostridium* species can produce 4 mol of H_2_ from 1 mol of glucose, which is a much higher amount than that produced by *Escherichia coli* and *Enterobacter* species (2 mol from 1 mol) (3). Currently, the bioproduction of butanol entails many problems due to the toxicity of this alcohol. One feasible strategy is to ferment biomass into butyric acid and then convert the downstream product into butanol (9). *C. butyricum* is preferred for butyric acid production owing to its higher productivity and the final concentration obtained (9). Meanwhile, *C. butyricum* is universally accepted as a good producer of 1,3-PD. Previous studies have indicated that *C. butyricum* can produce 1,3-PD from crude glycerol with a concentration of 76.2 g liter^-1^ and a productivity of 2.3 g liter^-1^ h^-1^ (10), showing promising potential applications for *C. butyricum* in industrial biotechnology.

Here, we present the first draft genome sequence of the *C. butyricum* type strain DSM 10702, obtained by using the Illumina HiSeq 2000 system, which was performed by the Chinese National Human Genome Center, Shanghai, China, with a paired-end library. The reads were assembled into 207 contigs by using Velvet (11). The genome annotation was performed by use of the RAST server (12). The G+C content was calculated by using the genome sequence.

The draft genome sequence of strain DSM 10702 is comprised of 4,596,811 bases with a GC content of 28.5%. There are 4,170 predicted coding sequences (CDS) together with 57 RNAs in the genome sequence of strain DSM 10702. According to the annotation, we have predicted 9 CDS responsible for xylose utilization, 9 CDS for l-arabinose utilization, 13 CDS for fructose utilization, and 17 CDS for glycerol uptake, suggesting that strain DSM 10702 has a wide substrate spectrum. There are 12 CDS that have been annotated as the genes related to butanol biosynthesis. The 1,3-PD operon, including the glycerol dehydratase- and 1,3-PD dehydrogenase-encoding genes, was also annotated. Moreover, the CDS responsible for the formation of organic acids, including butyrate, lactate, and acetate, were annotated. The pathways and key genes should be further investigated to eliminate side reactions to improve the production efficiency.

### Nucleotide sequence accession numbers. 

The whole-genome shotgun project has been deposited at DDBJ/EMBL/GenBank under the accession number AQQF00000000. The version described in this paper is the first version, number AQQF01000000.
